# Development and feasibility of a mindfulness-based dance/movement therapy intervention for chronic low back pain

**DOI:** 10.3389/fpain.2024.1281085

**Published:** 2024-04-10

**Authors:** Minjung Shim, Monica Gaydos, Natasha Goldstein-Levitas, Nicole Musalo, Nalini Prakash, Joke Bradt, Fengqing Zhang, Sarah Wenger, Adam Gonzalez

**Affiliations:** ^1^ Creative Arts Therapies, Drexel University, Philadelphia, PA, United States; ^2^ Psychology, Drexel University, Philadelphia, PA, United States; ^3^ Physical Therapy and Rehabilitation Science, Drexel University, Philadelphia, PA, United States; ^4^ Psychiatry and Behavioral Health, Stony Brook University, Stony Brook, NY, United States

**Keywords:** chronic back pain (CBP), non-pharmacological interventions, mindfulness—pain intervention, dance/movement therapy (DMT), mixed methods feasibility study

## Abstract

**Introduction:**

Responding to the need for innovative, multi-modal, non-pharmacological strategies in chronic low back pain (cLBP) care, this article presents the development and a mixed methods feasibility trial of a manualized Mindfulness-based Dance/Movement Therapy (M-DMT) program for cLBP. The 12-week program is designed as a group therapy, integrating mindfulness principles, creative/expressive dance and movement, and psychoeducational content focused on cLBP management. This holistic program seeks to cultivate nonjudgmental awareness of pain experiences, challenge maladaptive pain-related beliefs, enhance emotional well-being, foster social support, and promote effective coping strategies for the daily challenges associated with cLBP.

**Methods:**

The 12-week M-DMT intervention was administered to individuals with non-specific cLBP (*N* = 18, aged 51.7 ± 13.9 years, 72% female, 55% Black and 39% White). We assessed feasibility and acceptability through monitoring enrollment and retention rates, attendance, and adverse events. Moreover, we measured the intervention's credibility/expectancy, participants' perception of changes, and overall satisfaction. Additionally, we collected qualitative data, capturing participants' perspectives on the intervention's usefulness and perceived benefits. Specific benchmarks were established to gauge the successful feasibility and acceptability of the program.

**Results:**

The adherence rate stood at 80%, with a perfect retention rate of 100%. The study successfully met the benchmarks for treatment acceptability and satisfaction criteria, with 61% of participants reporting “feeling better” or a “great deal better” after the intervention. No adverse events were observed. Participants found the intervention enjoyable and reported that it provided effective tools for cLBP and related symptoms. Notably, participants reported a decrease in fear-avoidance behaviors, increased motivation for physical activity, and a boost in self-efficacy for pain management.

**Discussion:**

These encouraging findings establish a strong basis for considering the M-DMT intervention as a promising approach for cLBP management, warranting further investigation in larger-scale studies.

## 1 Introduction

Chronic low-back pain (cLBP) is a major public health problem worldwide and the second leading cause of disability among U.S. adults (1, 2). cLBP can impact various aspects of an individual's functioning and significantly influence their quality of life. Conventional management protocols for cLBP, such as opioids, nonsteroidal anti-inflammatory drugs, spine surgery, and nerve blocks, are associated with limited effectiveness and potential side effects, including opioid abuse and overdose deaths (3–5). As a result, there is an increasing trend towards the use of complementary and integrative pain management approaches (6). Between 80%–90% of LBP patients complain of non-specific low back pain. However, since the underlying pathoanatomical causes are not yet determined, the development of effective therapies is limited (7).

A review of clinical trials by the National Center for Complementary and Integrative Health reported promising effects of various complementary health approaches to pain management including mindfulness- and movement-based interventions, such as meditation, yoga, and tai chi (8). Mindfulness-based interventions (MBIs) were originally developed for and are consistently being used by individuals coping with various types of pain (9). Mindfulness is conceptualized as involving an intentional regulation of attention to and awareness of the present moment, as well as nonjudgmental acceptance of sensations, thoughts, and/or emotional states (10, 11). Thus far, studies have shown that MBIs, such as meditation and mindfulness-based stress reduction, may have significant effects on the reduction of back pain and disability (12–17). Potential therapeutic mechanisms of MBIs for cLBP include increasing body awareness, pain acceptance and tolerance as well as improving stress symptoms and depression (18). Nevertheless, like other cognitive-based approaches such as cognitive-behavioral therapy and psychoeducation, most MBIs primarily focus on increasing awareness and altering attention and thought processes, with limited emphasis on physical movement (19–21). Yoga, tai chi, qi gong, or walking meditations may be useful mindfulness-based movement practices for individuals with cLBP (22–24); however, these practices utilize specific poses or movement sequences that are prescribed and led by the instructor rather than movements that are creative, self-directed and self-expressive. The latter types of movement can activate intrinsic motivation for physical activity, foster a sense of agency and control, and directly address the emotional responses associated with pain through personally meaningful action, as opposed to prescribed, mechanical motion (25–28).

Additionally, mindfulness-based movement interventions (e.g., yoga, tai chi) as well as physical therapy, do not directly address specific cognitive-evaluative and affective-motivational factors associated with the vicious pain cycle in cLBP (e.g., pain catastrophizing, fear, anxiety, depression). Research indicates that maladaptive/hypervigilant attentional processes and anxiety-driven responses to pain hamper the necessary physical engagement for recovery, reinforcing the persistence of pain and disability—“fear-avoidance cycle” (29, 30). Gradual exposure to avoided physical activities while addressing pain-related fear is recognized as a fundamental non-pharmacological intervention to mitigate cLBP (31, 32). Given the critical importance of maintaining regular physical activity in the management of cLBP, it is imperative for interventions targeting cLBP to adeptly address maladaptive pain cognitions associated with the fear-avoidance cycle and foster intrinsic motivation for physical activity.

Dance/Movement therapy (DMT) is “the psychotherapeutic use of movement to promote emotional, social, cognitive, and physical integration of the individual, for the purpose of improving health and well-being” (33). DMT utilizes creative processes, self-expression, relaxation techniques (e.g., breathing and imagery-based exercises), movement improvisation, and interactive activities (e.g., mirroring, role play, group dance) as key methods to promote physical functioning, psychological health, self-efficacy, and social connectedness. Further, psychoeducation and cognitive processing are an integral part of DMT, enabling individuals to integrate corrective feedback and insights gained from somatic experiences into their cognitive awareness, thereby promoting positive psychological and behavioral changes (34).

DMT can be particularly effective in addressing psychosocial issues associated with cLBP as well as breaking the fear-avoidance cycle and promoting pain resilience (35, 36). Specifically, DMT utilizes in-vivo exposure to movement, such that spontaneous engagement in movement is encouraged and personal limitations for physical activity are respected. DMT's person-centered, self-directive approach to movement encourages individuals to activate intrinsic motivation for movement and provides an opportunity to re-evaluate personal capacity for physical activity (37). Experiencing the positive effects of movement provides evidence in real-time, which disconfirms and corrects catastrophic pain interpretations and fear-avoidance beliefs, thereby motivating continuous physical activity engagement (25, 35). Further, DMT provides a creative way to express and process negative emotions associated with chronic pain experience (e.g., depression, anxiety, anger, feelings of isolation) while promoting a range of positive emotions (e.g., happiness, joy, relaxation, social belonging), which can stimulate the release of endorphins, a body's natural pain reliever (38). Through accurate appraisal of one's physical efficacy for physical engagement and the pain-movement relationship, coupled with the positive emotions engendered by DMT, individuals gain confidence and further motivation to engage in more strenuous physical activity. Occasionally, despite the possibility of exacerbating pain, individuals are willing to persist and continue, recognizing the overarching benefits that these activities bring. From a cognitive standpoint, participating in creative dance, which involves exploring movement across different spatial and temporal dimensions, as well as qualities, while also engaging multiple senses, enhances cognitive flexibility and expands an individual's ability to focus. This, in turn, enhances the development of coping strategies and serves to counterbalance the obsessive focus on pain (39, 40). Socially, DMT actively utilizes group therapy factors to promote social connectedness and interpersonal competency (41). Furthermore, engaging in experiential learning plays a pivotal role in fostering a sense of control, thereby empowering individuals and encouraging resilience to maintain self-management (42). This process promotes an active role in one's own learning, facilitating a deeper understanding of oneself and enhancing the ability to effectively manage challenges. As such, M-DMT includes the benefits of physical activity, group psychotherapy, mindfulness training, and an art-based intervention in a unified practice.

Growing evidence supports the positive effects of DMT on various physical and mental health outcomes (e.g., depression, anxiety, mobility, body image, quality of life) (41, 43, 44). Several studies have explored the potential of DMT in various chronic pain management contexts (25, 28, 45, 46). However, the current body of research lacks randomized controlled trials (RCTs) and often exhibits limited methodological rigor. This includes the absence of standardized treatment manuals/protocols and a lack of evaluation of treatment fidelity, which collectively contribute to the insufficient evidence supporting the efficacy of DMT for chronic pain management. Furthermore, while evidence regarding the effect of mindfulness on cLBP has been established (47, 48), and fundamental mindfulness principles are inherent in DMT theories, only one study has, to date, explored the integration of two approaches within the context of cLBP care (46). Building upon prior research indicating the role of DMT in addressing maladaptive pain cognitions and activity avoidance, we hypothesized that teaching and incorporating mindfulness skills directly and explicitly (such as focusing on the present moment, one's body, and the experience of pain, alongside non-judgmental acceptance) within the framework of DMT could further enhance the treatment's effect on tackling fear-avoidance cycle. In light of this hypothesis, we developed a comprehensive manualized mindfulness-based dance/movement therapy (M-DMT) program tailored specifically for individuals with cLBP. This strategic integration aimed to synergistically potentiate and maximize the positive outcomes attributed to each modality. The aim of this study, therefore, was to test the acceptability, feasibility, and credibility of the M-DMT protocol in a sample of participants with cLBP.

## 2 Methods

### 2.1 Study design

This feasibility study used a mixed methods convergent design, in which qualitative and quantitative data were collected simultaneously, followed by the integration and comparisons of these multiple data sources (49). We merged the quantitative and qualitative findings during the interpretation or discussion stage to inform one another and to support or refute the research questions (50).

### 2.2 Participants

We recruited a convenient sample of 18 individuals with cLBP from two urban community health centers in Philadelphia. Participants were recruited through flyers, email blasts, referrals from primary care providers, and social media advertisements. Inclusion criteria were: (a) 18+ years of age; (b) current non-specific cLBP that has persisted at least 6 months; (c) a minimum of 3/10 on the numeric rating scale for pain over the past week; (d) if taking pain medication(s), the dosage must be stabilized for a minimum of 3 months; (e) agreement not to seek additional therapies for the duration of the study beyond those already included in the current treatment regimen (new treatments prescribed by their physician were allowed); and (f) naïve to DMT. Exclusion criteria were a) pregnancy; (b) severe and/or progressive medical or neurological conditions or severe mental illness (e.g., psychosis, mania); (c) cognitive impairment; (d) back surgery in the last 6 months; (e) back pain attributable to a recognizable, known specific pathology (e.g., infection, tumor, osteoporosis, fracture, structural deformity, inflammatory disorder, radicular syndrome, or cauda equina syndrome); (f) being wheelchair bound or unable to move without assistance; and (g) back pain-related compensation or litigation. Ethical approval was obtained from Drexel University's Institutional Review Board. Informed consent was obtained from all study participants.

### 2.3 The intervention

Mindfulness-based Dance/Movement Therapy (M-DMT) is a structured 12-session, 90-min/session, weekly program that was innovatively developed by the first author. (MS) This program is grounded in the biopsychosocial model of chronic pain, DMT theories, mindfulness, and psychological resilience literature (Table 1). The development of the M-DMT program by the first author is a significant contribution to the field and has been fully disclosed in line with ethical guidelines. The intervention was delivered by a board-certified dance/movement therapist NGL and was instructed to follow the manualized protocol. The overarching goal of this program is to offer mind-body based self-care tools for cLBP management and build individual's resilience resources to support overall health and wellbeing. The essential components of the M-DMT program include (1) active participation in both structured and spontaneous dance/movement-based exercises; (2) psychoeducation addressing physical and psychosocial dimensions of cLBP management and fostering healthy coping strategies; (3) acquisition of relevant mindfulness principles and techniques tailored to cLBP care; (4) self-directed learning and self-care practices; and (5) cultivation of social support networks. Psychoeducation topics and content were derived from chronic pain and DMT literature as well as guidelines available from the National Institute of Health (NIH), the US Department of Health and Human Services (HHS), and the Center for Disease Control and Prevention (CDC). Self-directed learning is facilitated by the utilization of a person-centered, non-directive approach to movement which encourages individuals to activate intrinsic motivation for movement and provides an opportunity to re-evaluate personal capacity for physical activity, thereby correcting maladaptive thought processes and beliefs around pain and physical activity. In addition, daily practice of mindfulness and movement-based pain management techniques is employed to support the development of self-care tools. A personal practice approach is used to encourage these between-session practices. The personal practice consists of various mindful movement sequences provided at the beginning of the intervention via YouTube links. Recognizing the importance of social support in a comprehensive approach to chronic pain management, the program actively integrates group therapy components to cultivate a therapeutic relationship and foster social connectedness among participants. Furthermore, we provided participants with a participant handbook containing a variety of Supplementary Materials (e.g., meditation and mindful movement scripts, descriptions of mindfulness concepts, and links to online mindfulness resources to support their self-directed learning.

**Table 1:** M-DMT 12-week program content.

Modules	Wk	Session topics	Main activities
I. Increasing self-awareness & positive attitude towards self	1	Introduction and rapport building	• Introduction to the program and group members, group agreement • Semi-guided movement warm-up and interactive group dance/movement • Introduction to the basic principles of mindfulness and DMT and establish a daily mindfulness practice
	2	Tuning into the body: mind-body connection	• Mindful awareness: body scanning and noticing bodily sensations • Exploring body image through mindful art: self-portrait or self-sculpture • Connecting to self through mindful breathing: 3-dimensional breathing, imagery-based breathing exercises, breath-guided dance/movement • Learning the “Connection dance” (independence study routine)
	3	Self-compassion and acceptance: making peace with your pain and self	• Moving meditation • Symbolic expression of pain through movement metaphor • Movement exploration of mindful attitudes (Nonjudging, patience, beginner's mind, trust, non-striving, acceptance, and letting go)
Self-feedback & reflection	4	Video review session I	• Nonjudgmental Approach • Watch video recordings of self from the previous sessions and self-reflection
II. Empowerment for self-care and symptom management	5	Self as an agent and expert	• Awakening senses: experience body as an active agent of perception by sensory-focused movement exercises (e.g., “I” see, ‘I’ hear, ‘I’ feel) • Choreograph/perform positive affirmative in action • “My pain management toolbox”: identifying personal strength and mindfulness strategies
	6	Developing coping skills and finding a balance	• Relaxation exercise (e.g., breathing and kinesthetic imagery-based relaxation) • Movement exploration of polarities, balance and modulation (Laban's effort theory-based practice) • Identify personal movement preference and habitual patterns
III. Cognitive-affective coping: managing thoughts and feelings	7	Attention modulation and articulation	• Exploration of Kinesphere: articulation and broadening attention scope and movement repertoires • Articulation of body parts through dynamic movement • Mindful refocus and attention modulation exercise
	8	Emotional awareness and Expression: Becoming smarter with feelings	• Notice and alter affective states through body and movement • Mindful art and movement expression: identify and express feelings through visual images and movement • Improvisational dance
	9	Self-compassion and acceptance: Integrating pain in the life trajectory	• Self-compassion and Acceptance: Acknowledging and befriending pain • Movement-based narrative • Loving kindness-focused authentic movement
IV. Relational coping	10	Relational intelligence and support-building	• Mindful awareness of nonverbal communication • Developing kinesthetic empathy (Mirroring, mover-witness practice) • Action-based group rapport-building exercises
Self-feedback & reflection	11	Video Review Session II	• Nonjudgmental Approach • Watch video recordings of self from the previous sessions and self-reflection
V. Maintenance & closure	12	Reflect & Integrate: Staying resilient	• Review the skills learned and develop individualized goals and plans • Establish sense of accomplishment and conclusion • Reinforce sense of community and hope: Group circle dance

Every session centered around distinct psycho-educational themes pertinent to cLBP management, interwoven with corresponding M-DMT techniques/experientials. A unique component of the intervention is the inclusion of a video self-review process. At weeks 4 and 11, participants review video excerpts from the preceding M-DMT sessions as a group. They are asked to observe various aspects of their own body and movement parameters (e.g., mobility, coordination, posture, affect state, expressivity, etc.) as well as interaction with others as a group. During the video review, the therapist encourages participants to cultivate nonjudgmental attention, acceptance, and self-compassion, fostering a discussion on self-awareness, maladaptive thinking, and nonjudgmental acceptance. Additionally, the therapist provides corrective feedback to foster adaptive responses among the group. Video self-review can help people with chronic pain gain insight into how their movements and postures contribute to their pain (51). It also enables people to track their progress and see improvements in their posture and movement habits. All sessions follow the structure of check-in, grounding meditation, a brief presentation on a psychoeducational topic (session theme), semi-guided movement warm-up, main activities linked to the session theme, cool-down, journaling, group discussion, and closing. There were two groups in total, each consisting of nine individuals.

### 2.4 Outcome Measures

To measure feasibility, we tracked the enrollment rate and attrition rate. We assessed the acceptability of treatment by tracking treatment adherence rates (i.e., the number of sessions attended). Our benchmark criterion for satisfactory acceptability was set at >75% of participants completing ≥9 sessions). We also used a treatment satisfaction survey. At the end of the intervention, a 5-point Likert scale survey was administered to measure participants' satisfaction levels (ranging from 1 = not very satisfied to 5 = very satisfied) with the treatment. An average score >3.5/5 was used as a feasibility criterion. Additionally, participants' experiences and perceptions of M-DMT intervention were explored through weekly feedback surveys (i.e., open-ended questions on most and least preferred elements of the session and suggestions for improvement). We recorded adverse events to monitor safety issues.

We assessed treatment credibility and expectancy quantitatively through the Credibility/Expectancy Questionnaire (CEQ) (52). CEQ was administered after session 1, after the full introduction of the treatment rationale and principles (53). The CEQ has an internal credibility rating of 0.81–0.86 and a reliability rating of 0.83. The first three items of the scale measure the credibility factor and the final three items measure the expectancy factor. In this study, we calculated credibility ratings by averaging the scores of the first three items of the CEQ, resulting in an overall credibility rating. For expectancy assessment, we selected one expectancy item from the CEQ, which prompts individuals to rate their anticipated improvement in cLBP on a scale of 0%–100%, using 10-point increments (i.e., “By the end of the therapy period, how much improvement in your cLBP do you think will occur?”). This expectancy rating maintains a high degree of face validity and has been the most widely utilized indicator of treatment outcome expectancy in psychotherapy research (53–56). Additionally, participants completed an 11-point numeric rating scale pre- and post-intervention to rate their average level of pain experienced over the past seven days. After completion of the M-DMT treatment, participants also completed the Patient Global Impression of Change (PGIC) (57). The PGIC is a seven-point scale with responses ranging from “no change” to, “a great deal better” to assess participants' perceived change in activity, emotion, quality of life, and symptoms directly or indirectly related to their pain. The PGIC shows strong psychometric properties and is included and recommended for use in clinical trials of the efficacy and effectiveness of chronic pain treatment by the Initiative on Methods, Measurement, and Pain Assessment in Clinical Trials (IMMPACT) (58). Specific criteria and benchmark goals for feasibility and acceptability outcomes are presented in Table 2.

**Table 2:** Feasibility and acceptability benchmark goals and evaluation.

Category	Data of interest and benchmark goals	Whether the goals were met or not (yes or no), QUAN and QUAL evidence
Feasibility of recruitment & enrollment process	1. Enrollment time: >6 people/month 2. Enrolment rate: ≥90% consenting 3. Feasibility of recruiting men: women to men ratio of 3:1 4. Source of recruitment: NCS 5. Reason for non-eligibility: NCS	1. Yes (Mean = 9 per month) 2. Yes (100%): *N* of people responded to the advertisement = 74; *N* of people screened = 43; *N* of people enrolled = 18 (Participants reported the mixed gender composition of the group, and the gender ratio were acceptable with men expressing desire to have more men in the group.) 3. Yes (Female = 13, Male = 5) 4. Flyers and e-newsletters (56%); approached by staff (28%); provider referral (11%); word of mouth (5%) 5. Unreachable (41%); scheduling conflicts (14%); specific back pain diagnosis (12%); childcare issues (4%); mobility restriction (1%); not naïve to DMT (1%); loss of interest (1%); and pain rating too low (1%)
Acceptability of treatment	1. Treatment adherence: ≥75% completing ≥9 sessions 2. Retention: ≤15% dropout rate 3. Reason for non-adherence, barriers to participation: NCS 4. Treatment acceptance and satisfaction: 4.1. Average score ≥3.5/5 on treatment acceptability rating scale 4.2. Exit interview (participants’ perception regarding the acceptability of the intervention contents, structure, and the interventionist)	1. Yes (80% completed ≥9 sessions) 2. Yes (0% dropout) 3. Time commitments outside the group (e.g., family or work responsibilities, doctor's appointments, and other planned activities), life stressors (e.g., unemployment or loss of loved ones), and health conditions 4.1. Yes [acceptability (*M* = 4.82); satisfaction (*M* = 4.53)] Participants reported overall satisfaction of the program contents and structure and expressed desire to continue.
Treatment credibility and expectancy	1. Treatment Credibility/Expectancy Questionnaire: ≥6/9 score on credibility items and >60% rating for expectancy Items	1. Yes (*M* = 7.78, SD = 1.34) for credibility items; (*M* = 70.59, SD = 21.35) for expectancy items)
Safety concerns	1. Weekly treatment safety and adverse events monitoring: NCS	No adverse event reported

NCS, no criteria Set; NA, not applicable.

Furthermore, we conducted a semi-structured interview to gather participants' qualitative feedback on various aspects of the program. Interview questions focused on their perceptions of the program's feasibility and safety, as well as their satisfaction with the delivery and comprehensibility of the content. We asked participants' perceptions of the helpfulness and enjoyment of the intervention, as well as their willingness to recommend it to others. Their opinions on the intervention's duration, dosage, and environment were sought, along with their evaluation of the utility of the handbook provided. This comprehensive feedback allowed us to assess participants' experiences and preferences and informed us regarding their likelihood of participating again.

### 2.5 Data analysis

In line with the study aims, quantitative data analysis was descriptive in nature. Means, standard deviations, effect sizes (i.e., Cohen's *d*) and associated 95% confidence intervals were reported. Qualitative data were analyzed using NVivo (59). We utilized a collaborative and iterative process of building the analytical framework and conducting data analysis (60). The PI MS and a research assistant (NP) developed a preliminary thematic framework by coding the first four transcripts using both deductive (based on *a priori* themes from the interview protocol) and inductive (open coding) approaches. This framework was then used by the other two coders NM and MG to analyze the remaining transcripts. The framework was consistently refined throughout the analytical process.

The mixed methods data analysis was conducted using the concurrent approach, where quantitative and qualitative data were initially analyzed separately and then the results were merged later. Qualitative data were used predominantly to increase the understanding of the quantitative results. Comparisons were made examining similarities and differences in results from the two data sets, noting salient points, where merging the data added to, confirmed, gave explanations for, or contradicted the results of each data set.

## 3 Results

To avoid unnecessary repetition, we have opted not to report detailed results separately for the quantitative and qualitative investigations. Instead, specific feasibility and acceptability benchmark goals and the evidence regarding whether the study met these goals are displayed in Table 2. The results of the mixed methods integration are presented below, offering a comprehensive understanding of the findings.

### 3.1 Participant characteristics

The mean age of participants was 51.72 (SD = 13.86), with 72.2% identifying as female. Among the participants, 55% were Black or African American, 38.9% were white, and 5.6% identified as Asian. Approximately 40% had experienced back pain for more than 5 years, and 44% reported using medication to alleviate their cLBP. For a comprehensive overview of the sociodemographic and pain-related attributes of the study participants (*n* = 18), please refer to Table 3.

**Table 3:** Sociodemographic and pain characteristics of study participants (*N* = 18).

Characteristics	*n* (%)
Age (years), mean (SD)	51.72 (13.86)
Female participants	13 (72.2%)
Ethnicity	
Hispanic	0 (0%)
Non-hispanic	18 (100%)
Race	
Asian	1 (5.6%)
Black or African American	10 (55.6%)
White	7 (38.9%)
Education	
High school	4 (22.2%)
Some college credit, no degree	6 (33.3%)
Bachelor's degree	5 (27.8%)
Master's degree	2 (11.1%)
Doctoral degree	1 (5.6%)
Marital status	
Single/never married	11 (61.1%)
Married/domestic partnership	5 (27.8%)
Widowed	2 (11.1%)
Employment status	
Working now	10 (55.6%)
Disabled for reasons other than	3 (16.7%)
Back pain	
Retired	4 (22.2%)
Other	1 (5.6%)
Duration of back pain	
3–6 months	3 (16.7%)
6 month—1 year	2 (11.1%)
1–5 years	6 (33.3%)
More than 5 years	7 (38.9%)
Baseline pain rating*	
3–4	5 (27.7%)
5–6	6 (33.3%)
7–8	4 (22.2%)
9–10	3 (16.7%)

^a^ Assessed by NRS ranging 0–10.

### 3.2 Enrollment rates

Out of the 74 individuals who responded to the study advertisement, 43 (58%) were screened. The remaining participants either could not be contacted or had lost interest in participating in the study. Eighteen people met the inclusion criteria and consented to participate in the study, successfully achieving the goal of an enrollment rate (≥90%).

When asked to provide suggestions for increasing participation by men in future studies, participants recommended, (a) designating a male “ambassador” from the program who can speak about the program and encourage other men to participate; (b) creating video demonstrations or promotional materials that feature men actively engaging in DMT activities; and (c) exploring the option of removing the term “dance” from the program description, as some participants expressed the belief that it might deter men from participating.

### 3.3 Retention, adherence, and adverse events

All participants completed the post-treatment data collection. Fourteen participants (80%) completed at least 9 out of 12 sessions (75%), successfully meeting the adherence goal. Reasons for inability to attend sessions included scheduling conflicts, life stressors (e.g., unemployment or loss of loved ones), and health conditions. Despite these challenges, the study achieved a high level of adherence, suggesting that the program was well-received and acceptable to participants. No adverse events were reported.

### 3.4 Treatment credibility and expectancy

During Session 1, participants (*n* = 16) completed the CEQ. The mean scores were 7.78, (SD = 1.34) for credibility items; and 70.59 (SD = 21.35) for expectancy, demonstrating that the study successfully met the goals (≥6 and ≥60% respectively).

### 3.5 Treatment acceptability and satisfaction

At the conclusion of the 12-week intervention, 17 people completed the treatment satisfaction and acceptability survey. We successfully met the benchmark goals (≥3.5/5) for both acceptability (*M* = 4.41, SD = .70) and satisfaction (*M* = 4.53, SD = .72) criteria, indicating a positive overall perception of the program's acceptability and satisfaction for individuals with cLBP.

During the exit interview, participants expressed satisfaction with all aspects of the M-DMT program (i.e., program content, implementation, demand and the dose, Supplementary Materials, the group facilitator, setting and the environment, and group gender composition). Participants found the program content easy to understand and said that it was delivered effectively. They said that the physical demands of the program were manageable, recognizing the program as a safe and feasible intervention for individuals with cLBP. Participants ascribed this to the adaptable/flexible nature of the program, especially with regards to expectations about movement quality and intensity based on an individual's capacity/limitation. Most of the participants felt that the duration of the program was appropriate while some wished that the sessions would have been offered more frequently. Participants also expressed their satisfaction with the group facilitator.

Components most enjoyed by participants included group check-ins, breathing exercises, enacting anchor phrases (i.e., sharing movement expression of one's positive affirmations for pain resilience with the group), learning about different movement qualities and the concept of *kinesphere* (i.e., space within the body's reach without stepping out of place), moving meditation, and the use of certain props (stretch band, scarves) to facilitate movement. Participants noted that the program's methods for conveying materials using a variety of modalities (imagery, craft, use of props, interactive movement-based learning) created opportunities for program content to be presented in interesting and new ways while making the content accessible and relatable to them. One participant noted, it “dipped everybody's toes into all of these different ways to channel our pain and stress”.

Participants perceived the program content as relatable/acceptable to all ages and genders and relevant to their back-pain issues; “The way it was structured was relevant to everybody's pain. It was relevant to us all”. Participants also noted that the program content was applicable outside of the group, as one participant said, “when I'm on the bus, wherever I am… I sit straight up, and I chill, and I just do some deep breathing.” Many of them said that despite some discouraging conditions and circumstances, they looked forward to attending the sessions, expressing a desire to continue the treatment. One participant stated: “I'm on disability and I don't come out often especially in this heat, but this was something I looked forward to coming to every week. I will miss doing this study.” The effectiveness of the handbook yielded mixed results; while some participants engaged with the relaxation and guided meditation scripts and accessed the additional resources provided via links, others predominantly relied on in-person sessions and utilized the handbook less frequently.

### 3.6 Perceived benefits

The findings from the PGIC revealed that 61.1% of the participants felt better or a great deal better after engaging in the intervention. Additionally, 27.8% reported moderate improvements, while 5.6% indicated no change. Pain intensity ratings suggested a small treatment effect of *d* = 0.26 [95% CI (1.67, 3.67)].

Qualitative data yielded valuable insights into the multifaceted benefits participants experienced as a result of their participation in the program. Participants expressed that the program equipped them with useful tools for pain management, leading to positive effects across various domains of functioning, including physical, emotional, cognitive, social, and behavioral aspects.

#### 3.6.1 Physical benefits

Participants acknowledged several physical benefits they experienced as a result of their participation in the program. They reported a notable reduction in fatigue and an increase in energy levels. Additionally, participants recognized improvements in their posture, which they attributed to engaging in movement imagery exercises and observing video footage of themselves during the sessions. One participant said, “when I sit or meditate, I use the balloon image and I'm able to straighten myself up… it helps relieving pressure off from my back too.”

Additionally, participants noted an increase in their range of motion and a decrease in their back pain. Participants acknowledged that their pain worsened as a result of decreased movement, and they observed a decrease in pain over time after increasing their level of physical activity. Many participants expressed an enhanced awareness of their movement patterns and recognized the importance of practicing mindful movement in managing pain.

*Now I stretch every morning to loosen up where it hurts, and I pay attention to my body movement when I do it. I was aware that movement will help it (back pain) but learned that you need to do the right kind of movement, mindful movement… When I go to axe throwing… I am mindful of my back while throwing the axe*.

Participants also reported an increase in their motivation to seek out and participate in a variety of ***physical activities***. Some participants reported notable improvements in their physical activity levels. They mentioned that the duration and distance of their walks increased, and they found themselves visiting the gym more frequently as well. One participant shared:

*Now I'm in the gym and in the pool, you know doing aqua therapy. These things all kicked off from this class. This class let me know that it might hurt a little but if you don't do something now, later it's going to hurt worse. So, I'm into movement now and looking for other types of things I can do. You know to keep on living*.

Lastly, participants shared that engaging in guided meditation and learning various breathing techniques had a profound impact on both their bodies and minds, inducing a state of relaxation. The movement experience itself provided a means to release physical tension and alleviate the mental stress that they had been experiencing.

#### 3.6.2 Emotional benefits

Numerous participants expressed that engaging in dancing and singing alongside the group led to an emotional catharsis that provided them with a valuable coping mechanism for their pain.: “Together, we talked, we laughed, we cried”. Additionally, participants reported improved mood, an increase in positive emotions, and peace of mind after participating in the program. One participant expressed: “It changed my mood. I was very happy the rest of the day and I felt, even if the word isn't happiness, I felt complete for that day”. Participants also looked forward to the M-DMT sessions because they were aware that their “stress level was going to decrease”. Additionally, participants discussed the usefulness of movement as a vehicle for self-expression and described how they learned to use movement to express feelings when words were not enough: “Sometimes words don't quite grasp everything you're feeling especially if you're trying to explain to someone else…. when we’d put a movement to it, that helped a lot. I feel it also helped me understand my pain.”

#### 3.6.3 Cognitive benefits

Participants attributed a number of cognitive benefits to the program including increased mindfulness, self-awareness, and self-reflection. Participants reported that the program taught them to “just really be aware of what's going on with my body,” which helped lessen their pain while performing day to day activities such as “gardening at home…or vacuuming my floor, or even just walking…I really haven't had any pain since I started these sessions because like I said, I was being more mindful of my movement.”

Several participants endorsed a change in their perspective towards life and their pain, developing a more positive attitude towards both their pain and themselves, and motivating them to continue with the treatment. One participant said:

*When I lost my ability to move and play and stuff, I became depressed. I became more involved in what I couldn't do. Not thinking of anything I could do. My woe is me….I can't I can't I can't. Coming here it says, you can you can you can you can. You just gotta show up and it happens a day at a time. You know, so it was a whole flip in consciousness that caused me to come every time*.

Another participant said:

*I think I feel more positive about my pain. Not glad that I have it but not looking at it as a bad thing or like as much as like a burden or that I have to you know make everyone make all kinds of accommodations*.

Participants expressed that deep breathing, movement meditation, and video self-reflection enhanced their ability to be more accepting, loving, and forgiving and less judgmental of themselves and their pain: “I learned to cope with it better and just accept that it is what it is and just do what I can”. “Keeping that judgement free….we were talking about forgiving ourselves, knowing our limitations and not be angry with ourselves for being in pain.” Participants also reported that within a few weeks into the program they had a better understanding of their pain and realized “that I was in a lot of pain, because I wasn't moving” and that “when you don't move, you hurt, everywhere all the time… Movement therapy is important.”

Additionally, participants shared that learning different movement techniques, taught them to refocus and distract from their pain making them more resilient to push past their pain. One participant shared: “When something's bothering, you just focus on doing something rather than being obsessed with the pain. So, now I would go out and just start working with my plants and during that time…my back wouldn't bother me.” Furthermore, participants acknowledged that the program helped them break free from the fear that resulted from physical pain. According to one participant:

*When you lay and you're just hurting, hurting, hurting, you become afraid and entangled in fear. You start thinking about death… it [the program] just opened up some things for me to make other steps to have a better life*.

Participants also discussed that the program increased their “confidence to try new things, helped me to loosen up more, to breathe more, move my arms more, just take up more space.” Another participant expressed that he “felt like I was about to fly. Like I'm flying while my back was still hurting.” Additionally, participants attested to learning and practicing mindfulness techniques they had never learned before, specifically “the movement aspect to mindfulness.” Participants learned to really think about “how to move…I do now that I have never done before” and also “learn how to breathe, it helps you just to calm down for a minute, relax, put away all your worries in the outside world.”

Finally, participants shared that partaking in freestyle movement enhanced their ability to be more creative in the way they managed their pain: “to figure out what I can do and how I can do it…. I make my own movement and hold it at the pace where it doesn't hurt.”

#### 3.6.4 Empowerment and transformation in pain management

Participants felt that the program helped them to take charge of their pain management, apply things they have learned from the program in their daily lives, and reshape their pain behaviors. A number of participants reported increased self-agency and motivation to actively cope with pain.

*I got the nerve to step out from this class into something else that is more positive, keeps me moving, is beneficial. Because all that laying….what is it, 4 years for me? I was stuck in bed…coming here kicked off one thing and then it kicked off another thing*.

Participants recognized that the program motivated them to engage in other health-promoting activities such as consuming a healthy diet: “I haven’t had fried chicken or fried fish in 12 weeks. It's either been baked or broiled. And it has me drinking more water and less juice and soda”.

In addition, participants shared that since they started the program, they were able to either reduce the dose of their pain medication or stop using it at all. Instead, they utilized the coping skills techniques they learned in the M-DMT program when they experienced pain and used medication as a last, not first, resort.

*Even if I did certain things or woke up uncomfortable, before I took anything I would just try to do some movements or whatever just to relax myself, and then if I had to take something later, I would take it later. But I didn't feel the need to take medication as soon as I woke up which I used to do*.

Finally, some participants discussed how easily they could adopt the mindfulness breathing and stretching techniques into their daily lives by way of meditation practices and/or adopting it as a lifestyle: “Like when I go out the building I'll stretch, I'll breathe, I do what she told me to do, look at a certain object, make sure you can focus on that thing”.

#### 3.6.5 Social benefits

Multiple participants expressed how the program helped them build trust and bond with each other over time. Participants felt “like a little family, little community” and “got closer and closer as time ended…we cared about one another”. Several participants also spoke about continuing to maintain their relationships even after the program ended and that their socialization increased even outside the program. Some participants acknowledged that moving and dancing with others gave them “the opportunity to learn from each other” because “everyone had a different take on (the exercises)”. Participants learned how to observe and identify emotions in each other, which made them more self-aware and more attentive to their feelings. Additionally, participants expressed that the program helped them realize that they were not alone in their pain and that they were able to share their experiences with other group members: “We all had some sort of pain, so we all understood. In a lot of ways, we were sharing with each other's certain experiences”.

Participants also reported feeling a heightened sense of empathy: “We felt each other's pain and were attentive to what my peers was going through…we were there for each other”. Participants also noticed that they were “more forgiving with other people” in the community and were able to apply skills they had learned in the program such as “taking breaths before reacting when other people irritate or upset me…I don't know what they're going through, take a step back”. Finally, participants attested to learning new ways of communicating through movement and one participant shared how she is now using movement to creatively communicate with her boyfriend: “Now we try *doing stuff* like explaining how our day went with a movement or like pain or anything like that. I found it really helpful as a communication tool. I really wasn't expecting that”.

#### 3.6.6 DMT as a distinct and valuable pain management approach

Participants acknowledged that many components of the M-DMT program were novel and different from any cLBP intervention they had experienced in the past. They described the M-DMT sessions as “a series of exercises and movements that are not only physical but psychological and perhaps even emotional”. They alluded to the fact that M-DMT was an alternate mode of engaging in physical activity and compared the M-DMT program to other practices they were familiar with such as yoga, physical therapy, gym sessions, and a regular dance class. Participants expressed their preference for M-DMT movements describing them as gentle, creative, holistic, and freestyle, allowing freedom of expression and flexibility in the way they chose to move (vs. static poses held in yoga), which increased their comfort levels. One participant said,

*I've done physical therapy before and that was so boring, and I dreaded going because I was just sitting there by myself doing the same thing over and over. But this was more entertaining and exciting, and I really looked forward to going to it*

Another participant compared the M-DMT to gym sessions: “The other thing that was different is (the facilitator) ain't working me like (gym instructor) works me… (the facilitator) is very flexible. You could either stand, you could sit, you can do this, you can do that.” Yet another participant compared the M-DMT sessions to a regular dance class:

*I thought it was going to be more like play music and here let's do this routine…. but I ended up liking this a lot more … I really liked the mindfulness component and working together rather than it being like an actual dance class*.

Additionally, participants expressed the novelty of “adding mindfulness to movement and adding movement to mindfulness,” and using movement as a tool for self-expression and communication. They enjoyed “communicating without sound, that was awesome”. One participant described the benefits of using props, imagery, and symbolic expression as therapeutic. Props such as the infinity band provided “an actual physical representation” of “an abyss going down” where they could toss their “emotions and things into the center pit, like getting rid of the stress and breathing in the positivity and getting rid of the stuff that we don't need on us anymore”. The same participant described how the artistic component of the program “tapped into the other side, not just the physical aspect of chronic pain but also the emotional side of things”.

Several participants also acknowledged that the program helped them come out of their shells, which made them “much more adaptable”, “feel uninhibited”, willing to try new things, and embrace taking risks. One participant shared:

*You're in so much pain you tend not to think about your breathing, you tend not to try new things or take risks. After the group I think everybody loosened up…for the first time ever, I was able to confidently come down to the shore for two days, whereas I might not have because of the physical limitations. I think that group just brought that out in people*.

#### 3.6.7 Perceived barriers and suggestions for optimizing program parameters

Participants described a number of personal and program-related barriers to their participation in the program. Some participants found it challenging to fully engage in the M-DMT due to their back pain or low energy levels. However, participants were reassured by the facilitator to only do movements they could handle and invited to modify as needed. A few participants expressed anxiety, self-consciousness, or resistance at the beginning of the program about dancing and expressing themselves in front of others: “It is kind of intimidating, especially when you don't know them and you're new to the group, it could be a vulnerable place to be in.” However, as the group progressed, this participant became more comfortable moving with others.

Participants identified challenges related to specific elements of the program, such as completing personal practice, gender composition, type of music, as well as implementation/delivery issues. Some participants felt there should have been more incentives offered or accountability for completing the personal practice. Regarding gender composition, one of the groups had only one male participant consistently attending the sessions. While the male participant himself did not perceive this as a barrier, some of the female participants expressed concern about comfort level in the group. One participant said, “I think I would have been very intimidated had it been me being the only female in the group.”

There were mixed responses regarding the video review sessions and music played during the sessions. While the majority of participants gained self-awareness from seeing themselves on the video recordings of the sessions, some expressed that watching themselves on the video made them feel uncomfortable due to prior body image or self-esteem issues. Regarding music, some participants wanted the music to have a more calming and meditative quality, while others thought that more upbeat music would make them feel more energetic at the end of the day.

Participants suggested using a customizable app, text messaging, or email reminders to notify them about the personal practice to increase accountability for personal practice. Another suggestion included having more YouTube videos of movements for home practice. Several participants expressed that they found the movement in the sessions “not rigorous enough.” and wanted “more dancing around the room” and for the movement to be “a little more intense.” Although they expressed wanting more vigorous movement, several participants felt that the word “dance” should be removed from the description of the program, explaining that they thought the word “movement” was more appropriate: “Because when you think of dance, you think of a real dance thing where there's ballet, hip-hop, whatever. It was more movement…movement just is not as intimidating.”

## 4 Discussion

This study tested the feasibility and acceptability of a newly developed complementary cLBP management protocol that combines mindfulness and DMT. The findings suggest that the 12-week group M-DMT program was well tolerated by individuals with cLBP and may be a promising treatment for improving physical and psychosocial outcomes related to cLBP. Using mixed methods offered a greater opportunity to examine the logistical feasibility and acceptability of the program parameters as well as the potential impact of the intervention. We met pre-established benchmark goals for most feasibility and acceptability parameters. Those who enrolled demonstrated a strong commitment to full participation, as evidenced by high session attendance as well as excellent follow-up rates with no dropouts. Participants found the duration, dosage, and frequency as well as the content and delivery of the intervention appropriate. These findings are significant in the context of chronic pain due to the high attrition rate often observed in clinical trials of mind-body intervention for chronic pain (61). The acceptability of the M-DMT intervention holds promise as it effectively tackles the specific challenges posed by chronic pain conditions, potentially reducing barriers to treatment adherence, and improving participant retention in clinical trials.

Qualitative findings enhanced our understanding of the feasibility and acceptability of the study logistics and intervention components while also shedding light on the perceived benefits of the intervention. All participants reported that they enjoyed participating in the M-DMT sessions and they felt that M-DMT was effective for managing their cLBP and associated symptoms. As the study progressed, participants experienced the benefits of M-DMT, which served as a significant motivational factor for their continued engagement. Through exit interviews and weekly qualitative feedback, participants expressed an enhanced awareness of the positive outcomes associated with the intervention, including improved physical and mental well-being. Research indicates personal positive experiences contribute to self-efficacy, fostering participants' motivation and confidence in engaging in mindful movement routines and reinforcing future practice (62, 63).

Participants also offered valuable insights and practical recommendations to enhance recruitment strategies and overcome engagement barriers in real-world clinical studies. Based on the findings, it is recommended to implement targeted advertising strategies to target the recruitment of male participants and address concerns associated with the inclusion of the word “dance” in the intervention title. Some individuals may be deterred by perceptions of the technical demands associated with the art form and gender-associated biases. A higher use of complementary and alternative medicine among females is evident in a number of clinical populations and men are frequently underrepresented in research studies focused on dance or mind-body based interventions (64, 65). Incorporating participants’ suggestions has the potential to optimize recruitment efforts by creating advertising campaigns that effectively resonate with male participants. Moreover, careful consideration of language and terminology in the intervention title can help alleviate apprehensions and encourage broader participation by dispelling misconceptions about the required technical expertise in dance. By implementing these adjustments, researchers can enhance the inclusivity and accessibility of their study, resulting in a more diverse participant pool and ultimately strengthening the external validity of their findings (66–68).

Another key finding of the study was the perceived benefit of the M-DMT intervention in managing cLBP and promoting psychological well-being. The majority of participants (61%) experienced a definite/considerable improvement that made a real and worthwhile difference in their activity limitations, symptoms, emotions, and overall quality of life in relation to their cLBP condition. Moreover, in alignment with earlier research, study participants reported increased motivation and willingness to actively engage in movement and exercise, attributed to the reduction in pain catastrophizing and fear-avoidance tendencies (25). These findings are particularly noteworthy in the context of the fear-avoidance model, which emphasizes the pivotal role of fear-avoidance beliefs in perpetuating and exacerbating chronic pain conditions. The observed decline in fear-avoidance tendencies, driven by the M-DMT intervention, suggests a significant disruption of the fear-avoidance cycle, potentially contributing to more effective chronic pain management strategies. It echoes previous research that underscores how DMT encourages self-directed movement engagement while fostering a heightened awareness of an individual's physical capabilities, ultimately resulting in increased motivation to participate in various other physical activities (25, 35).

Furthermore, the results regarding pain intensity suggest that the treatment yielded a modest reduction in pain perception. Nevertheless, it is crucial to recognize that in interventions targeting individuals with chronic pain, the primary objective is often not solely focused on pain reduction. Instead, emphasis is placed on fostering individuals' coping skills, promoting pain resilience, and facilitating positive changes in pain behaviors even if pain persists (69, 70). These outcomes may include strategies such as prioritizing non-pharmacological pain management strategies over the use of pain medication, with medication being considered as a last resort, as shown in our qualitative data. Moreover, participants demonstrated a willingness to persist through pain and actively engage in the intervention, even when certain movements had the potential to exacerbate their pain. This observation highlights the participants' decision to prioritize meaningful activities, understanding that the potential benefits of the intervention outweighed the temporary increase in pain. This concept of pain resilience coupled with pain *with* movement model (71), where individuals choose to persevere despite pain, lies at the core of their commitment to improving their well-being (72).

Drawing from the Mindfulness-to-Meaning Theory, Garland and Fredrickson (73) suggest that mindfulness practices can promote positive emotions that disrupt habitual cognitive patterns (e.g., movement exacerbates pain or may cause reinjury). This disruption allows for a broadened awareness and attention that incorporates a wider range of contextual information, enabling the construction of new and adaptive appraisals of self and the world. As a result, individuals' cognitive structures can be reconfigured, facilitating the ability to reappraise adversity and view these challenges as opportunities for psychological growth and resilience. The process of mindfulness-induced reappraisal can further amplify positive emotions by mindfully savoring the pleasant sensory aspects and deeper emotional significance of the situation. Shim and colleagues (35) have described this mechanism within the context of DMT, and the findings of the current study confirm the activation of these mechanisms. This indicates the potential of M-DMT to enhance pain resilience and overall wellbeing, which can be achieved by integrating mindfulness, reappraisal, and savoring, which, in turn, enhances natural reward processing. Natural reward processing refers to the brain's intricate system that responds to positive experiences and stimuli, releasing neurotransmitters such as dopamine, which are associated with feelings of pleasure and reinforcement (74). In the context of chronic pain, this reward system can become disrupted due to persistent discomfort, reduced physical functioning, and emotional distress that often accompany long-lasting pain conditions (75). By activating the brain's reward pathways, interventions like M-DMT contribute to improved emotional well-being, reduced pain-related distress, and increased overall quality of life for individuals living with chronic pain (76).

Lastly, the findings of this study underscore the profound social and emotional benefits of the M-DMT intervention. Participants reported a strong sense of community and trust, describing their group as a “family” that grew closer over time. Importantly, the intervention facilitated emotional awareness and empathy among participants, allowing them to connect with each other on a deeper level and offer support through shared experiences. The program also equipped participants with practical communication skills through movement, enabling creative expression and enhancing interpersonal relationships. These findings highlight the potential of M-DMT in fostering interpersonal connections, emotional well-being, and effective communication strategies among this population.

### 4.1 Limitations and future directions

This study had a small sample size, with a majority of participants being female (72%) and having at least some college-level education (78%). The participants had non-specific cLBP, were capable of engaging in moderate physical activity, demonstrating high motivation to participate in mindfulness and dance intervention. Furthermore, the study was conducted in urban health centers. Consequently, the generalizability of the findings may be restricted due to the specific characteristics of the sample. Further trials are necessary to assess the feasibility and impact of M-DMT in larger and more diverse samples of individuals with cLBP, including those with more severe pain symptoms and functional limitations. Additionally, it is important to note that this study did not incorporate a control group. Therefore, RCTs are warranted to systematically investigate the reported benefits. Moreover, while participants were encouraged not to start new treatments during the study unless recommend by their physician, data regarding concomitant treatment was not collected. The absence of this information limits our ability to account for potential confounding variables or to assess the full spectrum of treatment effects on cLBP outcomes. Future studies addressing these aspects would enhance the robustness and comprehensiveness of the research findings.

Future research endeavors should also aim to explore the outcomes of this intervention using both objective and subjective measures to provide a more comprehensive evaluation. For example, biometric data on physical activity levels or assessment of mobility performance through standardized tests (such as measuring range of motion via a goniometer or evaluating mobility using a timed up and go test) can provide concrete and objective data regarding the effectiveness of the intervention.

Additionally, there is potential in applying the elements of M-DMT to other forms of mindfulness or movement interventions, thereby expanding its reach and applicability. Furthermore, investigating the effectiveness of this intervention in other chronic pain populations could provide valuable insights and contribute to the development of tailored interventions for specific patient groups.

Despite these limitations, this study demonstrated strengths through the utilization of a mixed methods research design to systematically evaluate the feasibility and acceptability of an innovative mindfulness and dance/movement intervention for individuals with cLBP. By employing this approach, the study was able to provide a comprehensive understanding of the potential benefits and challenges associated with implementing such an intervention. These strengths contribute to the advancement of knowledge in the field and lay a solid foundation for further research in this area. The findings of this study have provided valuable insights into the positive attitudes exhibited by a traditionally challenging to engage group of individuals towards a comprehensive mindful movement intervention. Participants expressed optimism about the potential benefits of the intervention and reported significant improvements resulting from their active engagement in the program.

## 5 Conclusion

Our study represents one of the initial investigations into the feasibility and acceptability of a manualized M-DMT intervention for managing cLBP. The 12-week group intervention was shown to be feasible and demonstrated a range of perceived benefits for individuals with cLBP. The results suggest that the integration of mindfulness along with creative and expressive dance/movement-based techniques within a group therapy context offers individuals a holistic strategy for managing pain. It empowers them to take an active role in addressing their pain while enhancing their overall physical and psychosocial well-being.

## Data Availability

The raw data supporting the conclusions of this article will be made available by the authors, without undue reservation.
